# Bioactivity of *Carica papaya *(Caricaceae) against *Spodoptera frugiperda* (Lepidoptera: Noctuidae)

**DOI:** 10.3390/molecules16097502

**Published:** 2011-09-02

**Authors:** Salud Pérez-Gutiérrez, Miguel Angel Zavala-Sánchez, Marco Martín González-Chávez, Norma Cecilia Cárdenas-Ortega, Miguel Angel Ramos-López

**Affiliations:** 1 Departamento de Sistemas Biológicos, Universidad Autónoma Metropolitana-Xochimilco, Calzada del Hueso 1100, Col. Villa Quietud, México D.F. 04960, Mexico; 2 Centro de Investigación y Estudios de Posgrado, Facultad de Ciencias Químicas, Universidad Autónoma de San Luis Potosí, Av. Dr. Manuel Nava 6, Zona Universitaria, San Luis Potosí, SLP, 78210, Mexico

**Keywords:** fall armyworm, fatty acids, larvicide, insectistatic, control

## Abstract

The composition of a chloroform seed extract of *C. papaya* was determined by GC-MS. Nineteen compounds were identified, with oleic (45.97%), palmitic (24.1%) and stearic (8.52%) acids being the main components. The insecticidal and insectistatic activities of the extract and the three main constituents were tested. Larval duration increased by 3.4 d and 2.5 d when the extract was used at 16,000 and 9,600 ppm, respectively, whereas the pupal period increased by 2.2 d and 1.1 d at the same concentrations. Larval viability values were 0%, 29.2%, and 50% when the extract was applied at 24,000, 16,000, and 9,600 ppm, respectively; pupal viability was 42.9% and 66.7% at 16,000 and 9,600 ppm; and pupal weight decreased by 25.4% and 11.5% at 16,000 and 9,600 ppm. The larval viability of the main compounds was 33.3%, 48.5%, and 62.5% when exposed to 1,600 ppm of palmitic acid, oleic acid, or stearic acid, respectively.

## 1. Introduction

The fall armyworm *Spodoptera frugiperda* (J.E. Smith) is an insect that damages crops by consumption of foliage, and is the principal pest of maize in the tropical and subtropical regions of the Americas, where it seriously reduces crop yields. The economic effect is very high [1]. The insect also infests cotton, Bermuda grass, peanuts, and sorghum [2], and is a serious pest in Mexico [3]. Historically, various synthetic pesticides, including chlorinated hydrocarbons, organophosphates, carbamates, and pyrethroids have been used to combat this insect, but problems including development of resistance, damage to the environment, and killing of non-target organisms have become apparent. [4].

Plants produce a variety of insecticidal secondary metabolites that are less toxic to non-target species than chemical insecticides, and extracts of several plants have been used to combat *S. frugiperda*; an example is the triterpene azadirachtin from *Azadirachta indica* [5]. Some eudesmane-type sesquiterpenoids from *Pluchea sagittalis* have also been shown to deter larval feeding by *S. frugiperda*.

Franco *et al.*, evaluated the insecticidal effects of different powdered seeds against this insect, and found that the seeds of *C. papaya* caused high levels of larval mortality [6]. The contraceptive efficacy, reversibility, and toxicity of *C. papaya* seed products have also been investigated in rats and rabbits [7]. Moreover, powdered seed has been shown to have molluscicidal activity [8] and to combat the protozoan fish parasite *Ichthyophthirius multifiliis * [9]. As or seed extracts, the antifertility activity of a chloroform extract of *C. papaya* seeds has been evaluated in langur monkeys [10], but no study on the insecticidal effects of such extracts has yet appeared. Thus, our objective was to evaluate the effects of *C. papaya* chloroform seed extract on *S. frugiperda* and to determinate the composition of the extract.

## 2. Results and Discussion

Nineteen compounds in the seed chloroform extract of *C. papaya* were identified by GC/MS analysis, representing 85.81% of the extracted material (Table 1); the retention times ranged between 11.06 and 52.57 min. The main components were oleic acid (45.97%), palmitic acid (24.1%), and stearic acid (8.52%).

This composition differs from that reported by Puangsri *et al.* [11], who identified nine components in a petroleum ether seed extract of the “Batek Batu” *C. papaya* variety. The main compounds in their case were oleic acid (76.9%), palmitic acid (13.4%), stearic acid (4.6%), and linoleic acid (3.2%). Further, Figueroa *et al.* [12] found that an acetone extract of seeds of the “Mamey variety” of *C. papaya* contained seven components, among which the main constituents were oleic, palmitic, linoleic, and stearic acids, with relative percentage values of 76.75%, 12.89%, 4.11%, and 3.96%, respectively. The observed differences in composition may be attributable to the use of different varieties of *C. papaya* and different extraction solvents. The activities of the chloroform extract of *C. papaya *seeds are summarized in Table 2; the maximum insecticidal activity was observed at 24,000 ppm (0% of pupae formed). At 16,000 and 9,600 ppm, larval viability values were 29.2% and 50%, respectively, and pupal viability levels were 42.9% and 66.7% at the same concentrations. The LV_50_ was 10,198 ppm.

**Table 1:** Fatty acids of the chloroform extract of *C. papaya.*

No	Compound name	Retention time (min)	Peak area % *
1	Caprylic acid	11.06	0.06
2	Pelargic acid	15.21	0.11
3	Lauric acid	27.56	0.06
4	Myristic acid	34.95	0.51
5	Pentadecylic acid	38.41	0.15
6	Palmitoleic acid	41.03	0.56
7	Palmitic acid	41.99	24.1
8	Margaric acid	43.91	0.87
9	Oleic acid	45.25	45.97
10	Stearic acid	45.56	8.52
11	7,10-Octadecadienoic acid	46.21	0.17
12	Goindoic acid	47.46	1.37
13	Arachidic acid	47.73	1.19
14	Erucic acid	49.33	0.08
15	Beheric acid	49.55	0.64
16	Tricosanoic acid	50.36	0.13
17	Lignoceric acid	51.13	0.35
18	Pentacosanoic acid	51.86	0.06
19	Cerotic acid	52.57	0.07

* Values reported as a percentage of the total area.

**Table 2:** Mean (±SE) larvae and pupae duration, larvae and pupae viability and weight of *S. frugiperda* with chloroform extract of the seeds of *C. papaya *seeds.

Conc. ppm	Duration (d)		Viability (%)		Pupae weight (mg)
	larvae	Pupae	larvae	Pupae	
24,000	-	-	-	-	-
16,000	29.7 ± 0.67 *	14.7 ± 0.33 *	29.2 ± 9.48 *	42.9 ± 5.0 *	202.8 ± 4.72 *
9,600	28.8 ± 0.61 *	13.6 ± 0.30 *	50 ± 10.43 *	66.7 ± 16.67 *	240.6 ± 6.58 *
1,600	27.4 ± 0.73	12.8 ± 0.21	87.5 ± 9.03	95.2 ± 10.01	265.9 ± 7.13
0	26.3 ± 0.44	12.5 ± 0.17	91.67 ± 5.76	95.2 ± 8.33	271.8 ± 5.3
VL_50_			10.198 × 10^3^ ppm		

* Significant differences with the control, VL_50_ Larvae viability fifty.

Use of the extract at 16,000 and 9,600 ppm prolonged the larval phase by 3.4 d and 2.5 d, and the pupal phase by 2.2 d and 1.1 d, respectively. The pupal weight was reduced by 25.4% and 11.5% (compared to control weight) when the extract was given at the above concentrations. Thus, the chloroform extract was insectistatic against *S. frugiperda*.

Insecticidal activity of seed powder of the Hawaiian, Mamey, Maradol, and Yellow varieties of *C. papaya* against *S. frugiperda* was reported by Franco-Archundia *et al.* [6], who observed 90% mortality after 72 h in the presence of 33,600 ppm with the four varieties tested; mortality was 90% at 96 h when the Hawaiian, Mamey, and Maradol varieties were present at 22,400 ppm, and 75% when the Yellow variety was tested. Figueroa *et al.* [12] showed that an acetone extract of *C. papaya* seed, at 600 mg/15 mL (90,000 ppm) killed 60% of insects by 10 d. In both instances, insecticidal activity was less than observed when a chloroform extract was used.

An insecticidal bioassay of the main chloroform extract components of *C. papaya* seed (Table 3) showed that oleic acid reduced larval viability to 48.5% or 58.3% when present at 1,600 and 960 ppm for 15 days; viability values in the presence of palmitic acid were 33.3%, 50%, and 62.5% at 1,600, 960, and 400 ppm, respectively. Stearic acid reduced larval viability to 62.5% at 1,600 ppm. The LV_50_ values were 1,353.4, 989, and 2,176.5 ppm for oleic, palmitic and stearic, acids, respectively. These results indicate that individual fatty acids had higher insecticidal activities than did the chloroform extract, and palmitic acid was the most effective in this respect.

**Table 3:** Mean (±SE) larvae viability of *S. frugiperda* with oleic, palmitic and stearic acids.

Concentration (ppm)	Oleic acid	Palmitic acid	Stearic acid
1,600	48.5 ± 10.39 *	33.3 ± 9.83 *	62.5 ± 10.1 *
960	58.3 ± 10.28 *	50 ± 10.43 *	75 ± 9.03
400	70.8 ± 9.48	62.5 ± 10.1 *	87.5 ± 6.9
160	83.3 ± 7.77	70.8 ± 9.83	87.5 ± 6.9
0	95.8 ± 7.77	95.8 ± 7.77	95.8 ± 7.77
VL_50_	1.3534 × 10^3^ ppm	0.989 × 10^3^ ppm	2.1765 × 10^3^ ppm

* Significative differences with the control, VL_50_ Larvae viability fifty.

An insecticidal activity of oleic acid has been previously reported by Ramsewak *et al.* [13]; the LD_50_ value was 100 µg mL^−1^ when fourth instar larvae of *Aedes aegyptii *(Diptera: Culicidae) were exposed to the material. Growth inhibition (at the same oleic acid concentration) was evident when tests were performed using larvae of *Helicoverpa zea* (Lepidoptera: Noctuidae), *Lymantria dispar* (Lepidoptera: Lymantriidae), *Malacosoma disstria* (Lepidoptera: Lasiocampidae), and *Orgyia leucostigma* (Lepidoptera: Lymantriidae); the levels of inhibition were 85%, 91%, 80%, and 75%, respectively, after 1 week [13]. Rahuman *et al.* [14] found that oleic acid had LC_50_ values of 8.8, 9.79, and 7.66 ppm when used to kill *A. aegyptii*, *Anopheles stephensi*, and *Culex quinquefasciatus* (Diptera: Culicidae), respectively. No prior study has examined the insecticidal activity of fatty acids against *S. frugiperda*. The chloroform extract of *C. papaya *had significant insecticidal and insectistatic activity against *S. frugiperda*, indicating that such an extract may be used for pest control. The extract is selective, as a massive dose of 4,000 mg kg^−1^ would be required to kill mice.

## 3. Experimental

### 3.1. Plant Material and Extraction

The Maradol variety of *C. papaya* fruit was obtained from the “Central de Abastos” market of Mérida (Yucatán State, México). Seeds were shade-dried for a minimum of 15 days. Powdered seeds (1 kg) were extracted with chloroform (3.0 L), under reflux, for 4 h; the extract was cooled to room temperature and filtered. Solvent was removed under reduced pressure by rotatory evaporator and the extract was dried in a vacuum oven at room temperature for 12 h (yield, 7.2% by weight)

### 3.2. Larvae

Larvae of *S. frugiperda* were reared in plastic cages covered with iron mesh screens at 25 ± 2 °C, at 70 ± 5% relative humidity, with a light-dark cycle of 14:10 h. The artificial diet recommended by the International Maize and Wheat Improvement Center [15] was supplied.

### 3.3. Bioassays

The activity of the seed chloroform extract against *S. frugiperda* was explored at four extract concentrations, ranging from 1,600 to 24,000 ppm, and larval viability upon exposure to oleic, palmitic, and stearic acids was tested at four concentrations ranging from 160 to 1,600 ppm. Groups of 24 larvae were randomly selected to each concentration. The extract or fatty acid was admixed with the larval diet; all tests included a negative control (diet only). Food containing the chloroform extract or individual fatty acids was stored in acrylic glass vials (Bio-Serv Frenchtown, NJ, USA, catalog no. 9051) and held at room temperature for 24 h. Next, each larva of first instar of *S. frugiperda *was placed in the vials, and the vials were stoppered (Bio-Serv catalog no. 9049 stoppers). Pupae were weighed 24 h after pupation and transferred to fresh vials during development to the adult stage. Variables evaluated were the duration of larval and pupal periods; larval and pupal viability, pupal weights at 24 h; and larval viability in the presence of oleic, palmitic, or stearic acids [16]. Also, larval half-viability (VL_50_) values (the concentrations of additive at which 50% of larvae lived) were determined [17].

### 3.4. Chemicals

Oleic acid, palmitic acid, and stearic acid standards were purchased from Sigma-Aldrich (St. Louis, MO, USA).

### 3.5. Analysis of the Chloroform Extract

Fatty acid methyl esters were prepared according to the AOAC-IUPAC Method 969.33 [18]. Chloroform extract (90 mg) and 1 N solution of NaOH in methanol (4 mL) were placed in a round-bottomed flask, and the mixture was heated at boiling point with stirring for 15 min. Next, BF_3_-MeOH (5 mL, 15% w/w) were added and heating continued for 5 min. Iso-octane (2 mL) was added; the mixture was stirred for 5 min, more and extracted with hexane (2 mL). The organic phase was dried over anhydrous NaSO_4_. The fatty acid methyl esters were analyzed on an Agilent Technologies (Santa Clara, CA, USA) 6890N GC equipped with an HP-5MS column (30 m in length; 25 mm internal diameter; 0.25 μm film thickness) equipped with an Agilent EM 5973 detector, at 150 °C. The carrier gas was helium, at a flow rate of 1 mL/min; the split ratio was 2:1. The column temperature was initially 60 °C (for 3 min) and was gradually increased to 170 °C, at 3 °C/min; this temperature was held for 1 min. Next, the temperature was raised to 330 °C, at a rate of 10 °C/min; this temperature was held for 10 min. The injector temperature was 330 °C and 1 µL of organic phase were injected by duplicate. The identification of the components was confirmed by comparison of the retention indices with those of authentic compounds and with the NIST 02 library.

### 2.6. Acute Toxicity Assessment

The extract was orally administered at dose of 4,000 mg kg^−1^ to three mice of each sex [19]. After dosing, animals were observed under open-field conditions for 72 h. The number of deaths and all signs of clinical toxicity were recorded during the first 8 h post-dosing, and next daily, for 3 days.

### 2.7. Statistical Analysis

Statistical analysis employed was analysis of variance (ANOVA) followed by application of the Tukey test. A *p* value <0.05 was considered significant. LV_50_ values were calculated by Logit analysis, employing SYSTAT 9 software [20].

## 4. Conclusions

A chloroform seed extract of *C. papaya* (Maradol variety) had insectistatic and insecticidal activities against *S. frugiperda. *Among the identified constituents palmitic acid was the most active agent, followed by oleic and stearic acids. The low toxicity of the extract, and its effectiveness in terms of insect control, are all valuable attributes. Moreover, the development of an insecticide based on papaya seeds, which have traditionally been considered a waste product, could have commercial benefits.
